# Integration of clinical data with scanned ECGs using deep learning methods for stroke risk prediction in Indian patients with atrial fibrillation: evidence from the KERALA-AF study

**DOI:** 10.1016/j.lansea.2025.100715

**Published:** 2026-01-12

**Authors:** Qinkai Yu, Jinbert L. Azariah, Z. Sajan Ahmad, Rajappan Anilkumar, Peter Calvert, Yang Chen, Yalin Zheng, Yanda Meng, Bahuleyan Charantharayil Gopalan, Gregory Y.H. Lip

**Affiliations:** a Department of Computer Science, University of Exeter, United Kingdom; b Liverpool Centre for Cardiovascular Science at University of Liverpool, Liverpool John Moores University and Liverpool Heart & Chest Hospital, Liverpool, United Kingdom; c Department of Eye and Vision Science, University of Liverpool, United Kingdom; d Department of Cardiology, Lifeline Heart Institute, Lifeline Hospital, Adoor, Kerala, India; e Aster Cardiac Sciences at Aster Medcity, Kochi, Kerala, India; f Ananthapuri Hospitals and Research Institute, Chacka, Trivandrum, Kerala, India; g Global Institute of Public Health, Chacka, Trivandrum, Kerala, India; h Bioengineering Program, Biological and Environmental Science and Engineering Division (BESE), King Abdullah University of Science and Technology (KAUST), Thuwal, 23955, Saudi Arabia; i Department of Clinical Medicine, Aalborg University, Aalborg, Denmark; j Department of Cardiology, Lipidology and Internal Medicine with Intensive Coronary Care Unit, Medical University of Bialystok, Bialystok, Poland

**Keywords:** Atrial fibrillation, Stroke, Multimodal deep learning, Artificial intelligence, Paper-based electrocardiogram, Clinical data integration, Risk stratification, South Asia

## Abstract

**Background:**

Stroke risk stratification in patients with atrial fibrillation (AF) is challenging, particularly in under-represented South Asian populations. The use of a multimodal deep-learning artificial intelligence (AI) model, which integrates clinical data with widely available paper electrocardiogram (ECG) images, represents a novel predictive approach that has not previously been validated in this population.

**Methods:**

This study used data from the prospective KERALA-AF registry, the largest prospective AF study in South Asia. We developed a multimodal deep-learning AI model to predict incident stroke within one year by combining tabular clinical data with scanned paper ECGs. We benchmarked its performance (AUC) against machine learning (ML) models using only clinical data and the CHA_2_DS_2_-VASc score.

**Findings:**

Of 631 patients included (mean age 64.4, SD 12.9; 54.2% female), 25 (4.0%) experienced a stroke within one year. The multimodal deep learning AI model incorporating ECG data achieved the highest discrimination (AUC 0.816, 95% CI 0.704–0.914), substantially outperforming the CHA_2_DS_2_-VASc score (AUC 0.666) and all compared machine learning models trained on clinical data alone. Permutation analysis showed the scanned paper ECG images contributed 57.1% of the model's predictive signal, which boosted the model's performance significantly.

**Interpretation:**

Integrating scanned paper ECGs with clinical data via deep learning methods significantly enhanced 1-year stroke clinical risk prediction in South Asian AF patients. This study demonstrates the value of using multimodal AI with readily available, non-digital data in improving clinical risk stratification beyond current approaches based on clinical risk factors alone.

**Funding:**

Kerala Chapter of Cardiological Society of India.

**Research in context**

**Evidence before this study**

Stroke risk prediction in patients with atrial fibrillation (AF) has traditionally relied on clinical risk scores such as CHA_2_DS_2_-VASc, which show only modest discrimination and were largely derived from Western populations. Several machine-learning–based models using structured clinical or electronic health record data have demonstrated incremental improvements over traditional scores, including a small number of studies from East Asia and South Asia. However, these models have been restricted to tabular clinical variables and have not incorporated imaging data. Deep learning approaches using electrocardiogram (ECG) data have shown promise for arrhythmia detection and cardiovascular risk stratification. In South Asian settings, where paper-based ECGs remain widely used in routine clinical practice. There is a paucity of evidence on whether scanned ECG images can be leveraged alongside clinical data to improve stroke risk prediction.

**Added value of this study**

Using data from the KERALA-AF registry—the largest prospective atrial fibrillation registry in South Asia with recruitment from 53 centres across Kerala, India—we developed and validated a multimodal deep learning model that integrates scanned paper ECG images with routinely collected clinical data to predict 1-year stroke risk. Compared with the CHA_2_DS_2_-VASc score and multiple conventional machine-learning models trained on clinical data alone, the multimodal model achieved superior discrimination for 1-year stroke prediction. We further quantified the relative contributions of each modality, showing that scanned ECG images provided substantial and complementary predictive signal beyond clinical variables. Our findings establish the feasibility and added value of multimodal AI models that leverage routinely available, low-resource data sources in real-world South Asian healthcare settings.

**Implications of all the available evidence**

Taken together with existing evidence, our findings suggest that stroke risk stratification in AF can be meaningfully improved by moving beyond traditional clinical risk scores and unimodal clinical data. In regions such as South Asia, where digital ECG infrastructure may be limited, the ability to utilise scanned paper ECGs substantially broadens the applicability of advanced AI-based risk prediction. Future studies should focus on external validation in larger and more diverse cohorts, as well as on evaluating the clinical utility and implementation of multimodal AI models in prospective workflows.

## Introduction

Atrial fibrillation (AF), the most common sustained cardiac arrhythmia encountered in clinical practice, is a major public health concern associated with significant mortality and morbidity from stroke, heart failure, and dementia.^1^ However, the epidemiology of AF and the absolute risk of adverse outcomes such as stroke, exhibit substantial variation across different regions and ethnic groups.^2^

Stroke risk in AF is not homogeneous, and the more common and validated clinical factors have formulated stroke risk stratification schemes, the most commonly used being the CHA_2_DS_2_-VASc/CHA_2_DS_2_-VA scores.^3^^,^^4^ Nevertheless, such simple clinical scores have well-recognised limitations. For example, the CHA_2_DS_2_-VASc score does not account for several other potent risk factors, such as chronic kidney disease,^5^ specific AF subtypes,^6^ or informative electrocardiogram (ECG) features.^7^ These shortcomings are particularly pronounced in understudied populations. Most large-scale studies^8^, ^9^, ^10^ have also focused on Caucasian and East Asian cohorts, however, there is a paucity of data on stroke risk prediction specifically for South Asian populations. This underscores the need for accurate and inclusive risk prediction models tailored for patients with AF in this demographic.

Machine learning (ML) presents a powerful opportunity to overcome the limitations of traditional statistical models, which often assume linear relationships between variables thus losing stability as the number of predictors increases. By integrating a much larger and more diverse set of input factors, ML can build robust and nuanced predictive frameworks.^11^ While ML offers improvements over statistical scores, traditional ML algorithms (e.g., Random Forest, SVM) are generally restricted to structured tabular data and rely on manual feature engineering to process ECGs (e.g., measuring intervals), which may lose subtle non-linear visual patterns. A limited number of studies in South Korea,^8^ Japan,^9^ and South Asia^12^ have shown that ML models outperform the CHA_2_DS_2_-VASc score, but a critical research gap still persists.

In contrast, Deep Learning (DL) possesses the unique capacity for “representation learning” from unstructured raw data. This enables the automatic extraction of high-dimensional visual features from scanned images and their integration with clinical variables—a multimodal fusion task that traditional ML cannot perform directly. To the best of our knowledge, no prior study has developed a multimodal deep learning model that integrates clinical variables with imaging data from paper ECGs—a ubiquitous and diagnostically rich resource—to predict AF-related stroke in a South Asian cohort.

In this study, using data from the Kerala Atrial Fibrillation Registry (KERALA-AF), the largest prospective AF registry in South Asia, we developed a novel deep-learning AI model that combines clinical data with scanned paper ECG images to predict 1-year stroke risk. Second, we analysed the contribution of each data modality and identified the key predictive features that drive the model's performance.

## Methods

### Study participants

The KERALA-AF registry (trial registration details: CTRI/2017/10/010097) a prospective, multicentre cohort study of AF patients in the Kerala region of India, and is the largest prospective AF study in South Asia. The protocol and results of this study with one-year follow-up have been previously reported.^13^, ^14^, ^15^ During 2016–2017, 3421 AF patients were recruited from 53 independent centres.

### Inclusion and exclusion criteria

We included all patients enrolled in the KERALA-AF registry, excluding those who did not complete one-year follow-up. Patients without corresponding paper-based ECG scans were also excluded. Furthermore, ECG image data underwent rigorous manual visual quality control by trained research personnel. Scans were excluded based on specific ‘poor quality’ criteria, defined as: (1) Illegibility (severe blurriness or fading of thermal ink rendering P-QRS-T waveforms indistinguishable); (2) Incompleteness (missing leads or cropping errors failure to capture the standard 12-lead layout); (3) Obscuration (presence of handwritten annotations, stamps, or physical damage covering ECG traces); or (4) Severe Noise (excessive background artifacts distorting signal morphology).

### Data collection and outcome

Demographic characteristics, lifestyle factors, disease history, comorbidities, pharmacological and surgical treatments, imaging features, and laboratory parameters were collected at baseline, consistent with the KERALA-AF registry protocol. In addition, paper-based ECG tracings were scanned and incorporated as image data for analysis. The primary outcome of interest was incident stroke within one year of follow-up.

### Extracted features

We collected demographic characteristics (age, sex), comorbidities (diabetes, dyslipidemia, chronic kidney disease), lifestyle factors (smoking status), prior surgical history (mitral valve replacement), and AF-related characteristics (type of AF, treatment strategies including rhythm and rate control). Medication history included warfarin and diuretics. Other potential pharmacological predictors, specifically statins and antiplatelet agents (e.g., aspirin, clopidogrel), were initially assessed but were excluded from the final analysis due to missing data rates exceeding 50%, which surpassed our pre-specified quality control threshold. Imaging and laboratory features comprised left atrial size, systolic and diastolic blood pressure, body mass index (BMI), heart rate from ECG, aspartate aminotransferase (AST/SGOT), thyroid stimulating hormone (TSH), international normalised ratio (INR), total bilirubin, serum creatinine, and creatinine clearance. In addition to these tabular features, paper-based ECG tracings were scanned and incorporated as image data for model construction.

### Model construction

**Fig. 1:**
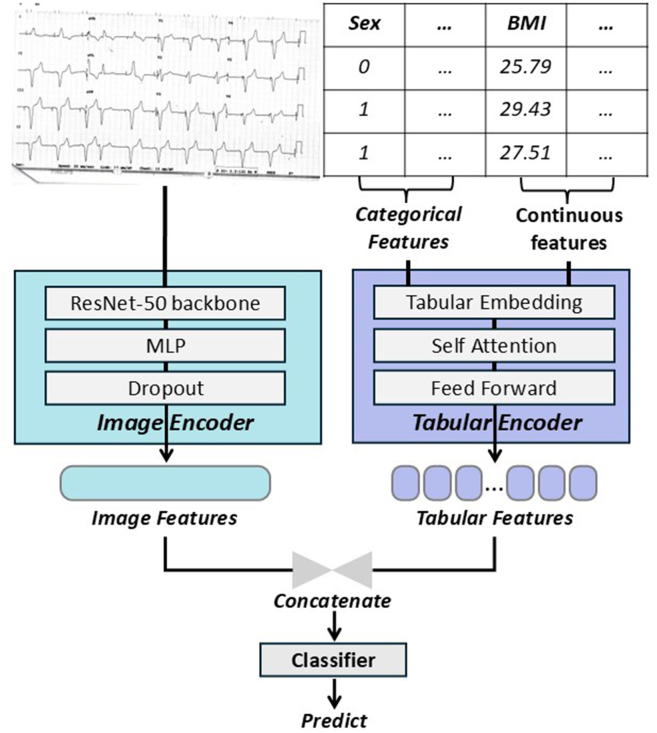
**Overview of the proposed multimodal deep learning architecture.** The framework consists of two parallel branches: (1) an **Image Encoder** (left) utilising a ResNet-50 backbone,^16^ MLP, and Dropout layers to extract features from ECG images; and (2) a **Tabular Encoder** (right) processing categorical (e.g., Sex) and continuous (e.g., BMI) clinical features via embeddings, self-attention mechanisms, and feed-forward networks. The resulting feature vectors from both modalities are **concatenated** and passed through a final classifier to generate the prediction.

We constructed a multimodal neural network integrating both clinical variables and ECG image data, as illustrated in Fig. 1. Clinical features (categorical and continuous) were encoded using a multi-layer feedforward network with batch normalisation and ReLU activation. Paper-based ECG scans were resized to 768 × 768 pixels, with pixel values rescaled to the 0–1 range. On-the-air Augmentation is conducted during training with random resizing and intensity variation (applied with 80% probability) to improve the model's generalisability. Paper-based ECG scans were processed through a ResNet-50^16^ encoder pretrained on ImageNet,^17^ followed by dimensionality reduction and projection into a 512-dimensional embedding space. The latent representations from the imaging and clinical feature branches were concatenated and passed through a classification head with dropout regularisation to predict the risk of one-year stroke.

Neural network weights were initialised using the method by He et al.^18^ Models were optimised with the AdamW^19^ optimiser, using a learning rate of 1 × 10^−4^ and weight decay of 1 × 10^−4^. The maximum number of training epochs was set to 100, with early stopping based on validation loss to reduce overfitting. Class imbalance was addressed using weighted loss functions.

For comparison, we implemented conventional machine learning classifiers trained solely on tabular features, including random forest (RF), logistic regression (LR), support vector machine (SVM), decision tree (DT), and k-nearest neighbours (KNN). The final analytic cohort (n = 631) was randomly split into two independent datasets using a 7:3 ratio. The Training Cohort (70%, n = 441) was used for model training and hyperparameter optimisation, while the Validation Cohort (30%, n = 190) served as the independent hold-out set to evaluate final model performance.

### Statistical analysis

We excluded variables with ≥30% missingness. For the remaining features, missing values were imputed using multivariate imputation by chained equations (MICE) with iterative random forests, consistent with previous KERALA-AF analyses.^12^ Categorical variables were rounded to the nearest integer after imputation to preserve their discrete structure. All variables included in the analysis had <30% missingness after preprocessing. Regarding data quality, the overall missing data rate for variables included in the final model was 9.07% (1202/13,251 data points). Specifically, the average missing rate was 9.67% for categorical features and 16.15% for continuous features.

For clinical tabular features, continuous variables were expressed as median (interquartile range) and compared between groups using the Kruskal–Wallis test, while categorical variables were expressed as counts (percentages) and compared using Fisher's exact test.

All models were implemented in Python (version 3.8.20) with PyTorch (version 2.4.1 + cu118), and training was performed on an NVIDIA RTX 4090 GPU.

Model performance was assessed by plotting receiver operating characteristic (ROC) curves and calculating the mean area under the curve (AUC) with 95% confidence intervals (CIs), estimated from 1000 bootstrap iterations. Additional performance metrics included accuracy, sensitivity, specificity, precision, recall, F1-score, and geometric mean (G-mean).

Predictive performance was benchmarked against the CHA_2_DS_2_-VASc score and five classic machine learning methods. Hyperparameters of the five conventional machine learning classifiers (RF, LR, SVM, DT, KNN) were optimised using grid search with five-fold cross-validation on the training set. Their performance was evaluated on the validation set, and AUC with 95% CIs was computed using bootstrap resampling. Feature importance for RF models was also extracted to provide interpretability. A two-sided p < 0.05 was considered statistically significant.

### Ethical approval and informed consent

The KERALA-AF registry study followed the principles in the Declaration of Helsinki and received ethical approvals from several ethics committees (Institutional Ethics Committee, Ananthapuri Hospitals and Research Institute; Institutional Ethics Committee, Sree Chitra Tirunal Institute of Medical Sciences and Technology; Ethics Committee, Lisie Heart Institute; Institutional Ethics Committee, Amrita Institute of Medical Sciences; Human Ethics Committee, Government Medical College, Trivandrum; Institutional Ethics Committee, Carithas Hospital, Kottayam; Institutional Ethics Committee, Sree Narayana Institute of Medical Sciences, Institutional Ethics Committee, Government Medical College, Calicut as well as by the Independent Ethics Committee of CSI–K). Since the data for this analysis were anonymised, no additional local ethical approval and informed consent was required.

### Role of funding sources

The Kerala-AF registry was supported by the Kerala Chapter of Cardiological Society of India through a one-time research grant No. CSI/IEC/2017. The funder did not play a role in the design or running of the study nor the analysis of results. No funding was received towards the analysis and writing of this manuscript.

## Results

### Cohort characteristics

The original KERALA-AF registry included 3421 patients. Of these, 560 patients without one-year follow-up were excluded. Among the remaining patients, those without corresponding paper-based ECG scans were further excluded, leaving 1813 patients. After quality control of scanned ECG images, an additional subset was excluded due to inadequate image quality, resulting in a final analytic cohort of 631 patients.

The median age was 65.0 years (IQR 55.8–74.0), and 46.8% were male. The prevalence of major comorbidities were diabetes (33.1%), dyslipidemia (40.1%), chronic kidney disease (10.0%), and history of mitral valve replacement (8.9%). Regarding AF type, 3.2% had paroxysmal AF, 0.6% had persistent AF, and 14.0% had permanent AF; AF subtype was not documented in the remaining 82.2%. Detailed baseline characteristics are summarized in Table 1.

**Table 1:** Baseline characteristics of the study cohort by 1-year stroke outcome.

Variable	Overall (n = 631)	Stroke-free (n = 606)	Stroke at 1 year (n = 25)	p value
**Demographics**				
Age, years	65.0 (55.8–74.0)	65.0 (55.5–74.0)	63.0 (58.0–70.0)	0.613
Male sex	295 (46.8%)	293 (48.3%)	2 (8.0%)	<0.001
BMI, kg/m^2^	24.4 (21.7–27.3)	24.3 (21.6–27.3)	26.0 (22.2–28.1)	0.190
**Medical history**				
Diabetes	210 (33.3%)	201 (33.2%)	9 (36.0%)	0.829
Dyslipidemia	253 (40.1%)	247 (40.8%)	6 (24.0%)	0.101
Chronic kidney disease	64 (10.1%)	62 (10.2%)	2 (8.0%)	1.000
Mitral valve replacement	56 (8.9%)	52 (8.6%)	4 (16.0%)	0.267
**Clinical examination**				
Systolic BP, mmHg	130.0 (120.0–150.0)	130.0 (120.0–150.0)	130.0 (117.5–150.0)	0.728
Diastolic BP, mmHg	80.0 (70.0–90.0)	80.0 (70.0–90.0)	80.0 (70.0–90.0)	0.782
Heart rate (ECG), bpm	90.0 (70.0–116.0)	90.0 (70.0–116.0)	100.0 (90.0–115.0)	0.025
Rhythm-control strategy	92 (14.6%)	89 (14.7%)	3 (12.0%)	1.000
Rate-control strategy	508 (80.5%)	487 (80.4%)	21 (84.0%)	0.800
Diuretics	297 (47.1%)	280 (46.2%)	17 (68.0%)	0.040
Warfarin	333 (52.8%)	316 (52.1%)	17 (68.0%)	0.153
**Laboratory values**				
AST (SGOT), U/L	30.0 (23.0–41.0)	30.0 (22.8–41.0)	32.0 (24.0–44.0)	0.461
TSH, mIU/L	2.1 (1.2–3.3)	2.1 (1.2–3.3)	1.2 (1.0–2.9)	0.262
Serum creatinine, mg/dL	1.0 (0.8–1.2)	1.0 (0.8–1.2)	0.9 (0.8–1.1)	0.252
Creatinine clearance, mL/min	57.4 (43.1–71.8)	57.6 (43.0–72.0)	56.4 (43.3–67.0)	0.955
**Echocardiographic parameters**				
LA size, mm	42.0 (37.0–47.0)	42.0 (37.0–47.0)	42.0 (38.0–46.5)	0.921
AF type: paroxysmal	20 (3.2%)	18 (3.0%)	2 (8.0%)	–
AF type: persistent	4 (0.6%)	4 (0.7%)	0 (0.0%)	–
AF type: permanent	88 (13.9%)	88 (14.5%)	0 (0.0%)	–

Data are median (IQR) or n (%), unless otherwise indicated. **Abbreviations**: AF, atrial fibrillation; AST, aspartate aminotransferase; BMI, body mass index; BP, blood pressure; ECG, electrocardiogram; LA, left atrium; SGOT, serum glutamic oxaloacetic transaminase; TSH, thyroid-stimulating hormone.

During the one-year follow-up, 25 patients (4.0%) experienced a stroke event. Compared with the non-stroke group, AF patients with stroke were more commonly female (92.0% vs 51.7%) had higher body mass index and heart rate, and were more likely to have undergone mitral valve replacement and to be receiving diuretic or warfarin therapy. Dyslipidaemia was less common among stroke patients, whereas other echocardiographic and laboratory parameters were comparable between groups.

### Model performance

**Fig. 2:**
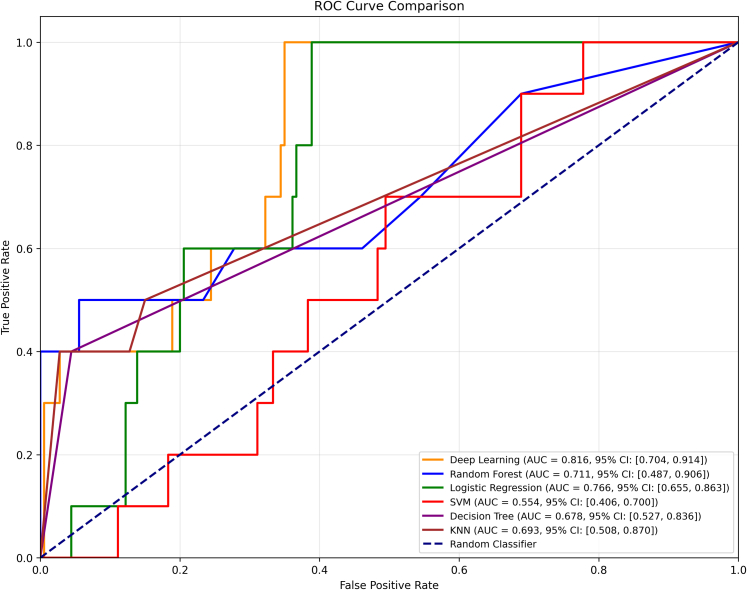
**Receiver operating characteristic (ROC) curves for multimodal deep learning and machine learning models in 1-year stroke prediction.** ROC curves compare the discrimination performance of the multimodal deep learning model (orange; integrating paper-based ECG images and clinical data) with traditional machine-learning models trained on tabular clinical variables only: random forest (blue), logistic regression (green), support vector machine (red), decision tree (brown), and k-nearest neighbours (purple). The dashed black line represents a random classifier (AUC = 0.50). The deep learning model achieved the highest AUC (0.816, 95% CI 0.704–0.914), outperforming all other approaches. AUC, area under the receiver-operating characteristic curve; CI, confidence interval; ECG, electrocardiogram; KNN, k-nearest neighbours; SVM, support vector machine. All ROC curves were derived from the same independent validation cohort.

As shown in Fig. 2, in the validation cohort (25/631 events; 4.0%), the multimodal deep-learning model showed the best discrimination with an ROC–AUC of 0.816 (95% CI 0.704–0.914), outperforming clinical tabular feature only baselines (logistic regression 0.766 [0.655–0.863], random forest 0.711 [0.487–0.906], k-nearest neighbours 0.693 [0.508–0.870], decision tree 0.678 [0.527–0.836], and SVM 0.554 [0.406–0.700]). At the validation-selected operating threshold, the multimodal AI model achieved sensitivity 0.30, specificity 0.994, precision 0.750, F1-score 0.429, G-mean 0.546, balanced accuracy 0.647, and MCC 0.458.

Among tabular baseline data, random forest delivered the highest F1-score (0.500) with sensitivity 0.40, specificity 0.989, and precision 0.667; logistic regression had higher AUC (0.766) but markedly lower precision (0.118) and F1 (0.197) owing to excess false positives (Table 2). These findings highlight that the multimodal deep learning provides superior discrimination, whereas operating-point trade-offs (e.g., F1 and G-mean) were influenced by thresholding under class imbalance, as expected in this low-event-rate setting.

**Table 2:** Performance of machine learning models and CHA_2_DS_2_-VASc–based approaches for 1-year stroke prediction.

Model	Sensitivity	Specificity	Precision	Recall	F1-score	G-mean	MCC	AUC	AUC 95% CI
**Multimodal deep learning model (Paper-based ECG and clinical data)**	0.300	0.994	0.750	0.300	0.429	0.546	0.458	0.816	(0.704–0.914)
Machine-learning models (clinical data only)									
Random forest	0.400	0.989	0.667	0.400	0.500	0.629	0.497	0.711	(0.487–0.906)
Logistic regression	0.600	0.750	0.118	0.600	0.197	0.671	0.176	0.766	(0.655–0.863)
Support vector machine	0.400	0.650	0.060	0.400	0.104	0.510	0.023	0.554	(0.406–0.700)
Decision tree	0.400	0.956	0.333	0.400	0.364	0.618	0.326	0.678	(0.527–0.836)
K-nearest neighbours	0.400	0.922	0.222	0.400	0.286	0.607	0.246	0.693	(0.508–0.870)
**CHA_2_DS_2_-VASc–based models**									
Logistic regression	0.000	1.000	0.000	0.000	0.000	0.000	0.000	0.666	(0.460–0.842)
Random forest	0.000	1.000	0.000	0.000	0.000	0.000	0.000	0.652	(0.513–0.790)
Support vector machine	0.000	1.000	0.000	0.000	0.000	0.000	0.000	0.523	(0.347–0.697)
Simple neural network	0.000	1.000	0.000	0.000	0.000	0.000	0.000	0.666	(0.460–0.842)

Data are presented as metric values rounded to three decimal places. Abbreviations: AUC, area under the receiver-operating characteristic curve; CI, confidence interval; MCC, Matthews correlation coefficient; G-mean, geometric mean; ECG, electrocardiogram. Performance metrics for all models were calculated using the same independent validation cohort.

Note: The multimodal deep learning model integrates Paper-based ECG with clinical variables, whereas all other machine-learning models use tabular (clinical) data only. CHA_2_DS_2_-VASc–based models use only the CHA_2_DS_2_-VASc score for prediction.

To statistically evaluate the performance improvements, we compared the AUC of our proposed Multimodal Deep Learning model against the baseline methods using DeLong's test. The analysis showed that our model significantly outperformed the SVM baseline (p < 0.001). Compared to ensemble and linear methods, our model achieved the highest numerical AUC, though the differences did not reach statistical significance (Random Forest: p = 0.12; Decision Tree: p = 0.31; KNN: p = 0.23; Logistic Regression: p = 0.38).

### Benchmarking against CHA_2_DS_2_-VASc

**Fig. 3:**
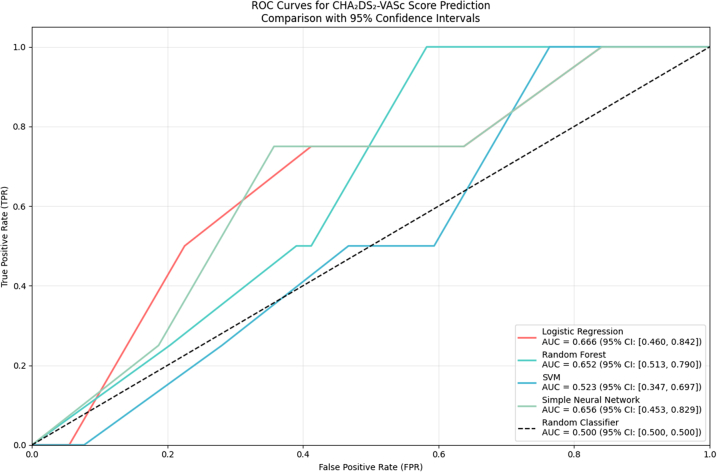
**Receiver operating characteristic (ROC) curves for CHA_2_DS_2_-VASc–based models in 1-year stroke prediction.** ROC curves illustrate the discrimination performance of four CHA_2_DS_2_-VASc–based classifiers: logistic regression (red), random forest (green), support vector machine (blue), and simple neural network (grey). The dashed black line represents a random classifier (AUC = 0.50). AUC values with 95% confidence intervals are shown in the legend. AUC, area under the curve; CI, confidence interval; SVM, support vector machine; ROC, receiver operating characteristic. All ROC curves were derived from the same independent validation cohort.

As shown in Fig. 3, using the CHA_2_DS_2_-VASc score as the sole predictor, discrimination was modest (ROC–AUC 0.666 [95% CI 0.460–0.842] with logistic regression; 0.652 [0.513–0.790] with random forest; 0.523 [0.347–0.697] with SVM; 0.666 [0.460–0.842] with a simple neural network).

As summarized in Table 2, using default decision thresholds, these models yielded zero sensitivity (and thus F1/G-mean/MCC = 0), reflecting the extreme class imbalance and the fact that thresholding was not tuned for this cohort. The ROC curves indicated non-zero separability (AUC ∼0.65), which remained substantially lower than the multimodal AI model (AUC 0.816).

### Feature importance and modality contribution

To interpret the model, we quantified (i) modality-level contribution by permuting each modality on the validation set and measuring the drop in AUROC, and (ii) clinical tabular feature importance by permuting each clinical variable while keeping the imaging branch unchanged.

#### Modality level

On permutation-based ablation, the ECG-image branch accounted for 57.1% of the total performance drop and the clinical tabular feature branch for 42.9%, indicating that ECG images contribute slightly more unique signal than the clinical tabular features, while both modalities were informative and complementary.

#### Clinical tabular feature importance (validation-set permutation)

**Fig. 4:**
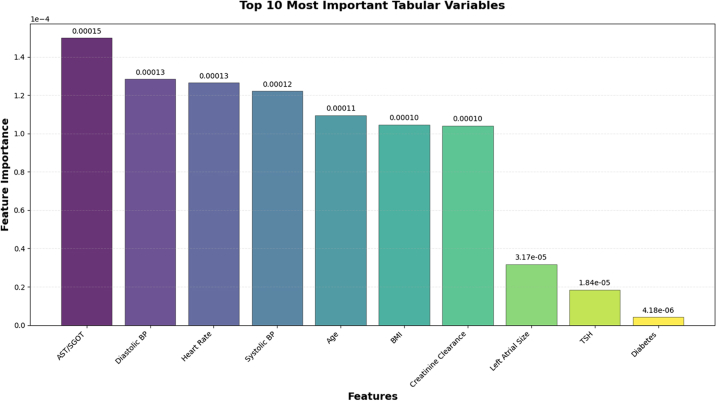
**Top 10 most important clinical tabular variables contributing to stroke prediction in the multimodal deep-learning model.** Bars represent permutation-based feature importance, quantified as the mean AUROC decrease when each clinical variable was permuted on the validation set while the ECG-image branch remained unchanged. Higher values indicate stronger predictive contribution. AST/SGOT, diastolic blood pressure, heart rate, systolic blood pressure, age, BMI, and creatinine clearance were the top contributors, followed by left atrial size, TSH, and diabetes.

As shown in Fig. 4, the highest-ranking contributors were AST/SGOT, diastolic BP, heart rate from ECG, systolic BP, age, BMI, and creatinine clearance; followed by left atrial size and TSH. Lower but non-zero contributions were observed for diabetes, sex, AF treatment strategies (e.g., rhythm control), serum creatinine, warfarin, diuretics, and mitral valve replacement. Dyslipidemia, smoking status, AF type indicator, and chronic kidney disease contributed negligibly in this model.

## Discussion

In this study, we developed a multimodal prediction model that integrates scanned paper ECG images with clinical tabular variables to estimate 1-year stroke risk in patients with atrial fibrillation (AF). We demonstrate that this multimodal approach achieves significantly better performance than classic models that rely on clinical data alone or CHA_2_DS_2_-VASc score, thereby offering a more effective pathway for accurate stroke risk stratification in this population.

Our model expands beyond prior methods by incorporating imaging-derived information and additional laboratory and treatment features. Compared with clinical tabular features-only baselines, the multimodal approach achieved higher discrimination (ROC–AUC 0.816), indicating that ECG images provide complementary signal to routine clinical data. Permutation-based ablation experiments suggested that the ECG branch contributed ∼57% and the tabular branch ∼43% of the overall performance boost, supporting the value of combining modalities.

It is also worth noting that the ECGs in this study were acquired from 53 diverse clinical centres using a heterogeneous mix of device manufacturers (e.g., GE, Philips, Schiller, BPL) rather than a single standardised model. Consequently, the input images exhibited significant variation in print layouts, background grids, and signal rendering. Despite this inherent variability in image characteristics, our multimodal deep learning model maintained high discrimination performance (AUC 0.816). This indicates that the algorithm is robust to device-specific variations and capable of extracting pathological features across different recording standards, which is essential for real-world deployment.

Regarding clinical feature importance, our model identified AST (SGOT) as a leading predictor of stroke risk. While AST is not a conventional component of stroke risk scores, this finding is consistent with prior machine learning analyses from the KERALA-AF registry,^12^ which also highlighted liver function markers as key prognostic features. This association likely reflects a ‘cardio-hepatic’ interaction, where elevated AST serves as a surrogate marker for hepatic congestion or hypoperfusion secondary to underlying heart failure or haemodynamic instability—conditions known to exacerbate thromboembolic risk. However, this finding should be interpreted with caution. AST is likely a marker of systemic disease severity and multimorbidity rather than a direct causal mechanism for stroke. Its high importance rank in this specific South Asian cohort may also reflect population-specific metabolic or hepatic characteristics that warrant further investigation.

Currently, there are limited studies on the application of machine learning/deep learning methods to predict stroke in Asian patients with AF. To date, these models have only outperformed clinical risk stratification using the CHA_2_DS_2_-VASc score in specific cohorts from South Korea, Japan, and South Asia.^8^^,^^9^^,^^12^ However, there are no prior reports on the use of imaging and clinical data to predict AF-related stroke in South Asian cohorts.

The observed 1-year stroke incidence was ∼4.0% (25/628) in this study's KERALA-AF cohort. This is lower than estimates from a multinational registry (RE-LY) spanning 47 countries, where Southeast Asia reports ∼7% 1-year AF-related stroke.^20^ Several factors may explain the differences, for example, case-mix and age distribution, treatment patterns, event adjudication and follow-up procedures, and importantly, sample attrition—our analytic cohort excluded patients without 1-year follow-up, without matched paper ECG scans, and with low-quality ECG images. These necessary exclusions improve data integrity for imaging analysis but introduce selection bias and somewhat limit generalisability.

We also recognise the modest event count (25 strokes), which increases statistical uncertainty, especially for threshold-dependent metrics and feature-importance rankings. The outcome was highly imbalanced (∼4% positives), so we adopted mitigation strategies during model training, including minority-class oversampling/stratified mini-batches and a class-weighted loss, to stabilise deep learning optimisation process and to avoid trivial solutions (i.e. resampling was applied to the training data only, never to validation/testing). Nevertheless, operating-point trade-offs (e.g., sensitivity–precision) still reflected the underlying imbalance. We therefore reported multiple complementary metrics (including G-mean and MCC) with bootstrap uncertainty to provide a more balanced assessment of performance. In terms of discrimination, while our multimodal model achieved the highest numerical AUC, the improvement over strong baselines (e.g., Random Forest, Logistic Regression) did not reach statistical significance (p > 0.05). This finding is likely attributable to the limited statistical power of DeLong's test, constrained by the small sample size of the independent validation cohort (n = 190) and the scarcity of stroke events (n = 10). However, unlike traditional machine learning models which often face a performance plateau due to fixed tabular features, deep learning architectures possess a superior capacity for representation learning. Consequently, our proposed approach demonstrates greater scalability, with the potential for the performance gap to widen as data volume increases, making it a more robust solution for future large-scale applications.

This work builds on prior KERALA-AF analyses^12^ that used clinical variables alone to predict 1-year outcomes. The previous analysis by Chen et al.^12^ had more stroke events (83 cases) and therefore greater statistical power. By contrast, our focus was to test whether adding ECG image information improves discrimination—despite fewer events—and to establish a feasible pipeline for leveraging real-world scanned (non-digital) ECGs.

Most AF-related stroke prediction tools rely on clinical scores such as CHA_2_DS_2_-VASc, which show modest discrimination across diverse cohorts.^20^^,^^21^ Subsequent machine-learning models using EHR tabular features have reported incremental gains but often remain constrained by the same information sources and class imbalance.^12^^,^^22^

In contrast, ECG-based deep learning has demonstrated strong signal for arrhythmia detection and cardiovascular risk stratification, yet prior studies typically used digital 12-lead waveforms and rarely targeted AF-related stroke specifically.^23^, ^24^, ^25^ Furthermore, while some technical studies benchmark performance only using ECG data,^26^, ^27^, ^28^ we did not employ a unimodal ‘ECG-only’ model for comparison in this study. In actual clinical scenarios, diagnosis and risk stratification are inherently holistic and invariably integrate ECG findings with patient history; relying solely on ECG traces without clinical context is clinically inadequate and rarely reflects real-world practice. Consequently, our primary objective was to demonstrate the incremental value of adding scanned paper ECGs to the current standard of care represented by clinical-only models. This approach mimics the physician's integrative workflow and focuses on the added value of multimodality, whereas evaluating the ECG as a standalone predictive tool would offer limited practical relevance. Multimodal deep learning frameworks combining imaging and clinical data improved prediction performance in other cardiovascular settings (e.g., heart failure, ACS), supporting the value of complementary modalities.^29^, ^30^, ^31^

Our work extends this literature in three ways: (i) this is, to the best of our knowledge, one of the first multimodal deep learning model in a South Asian AF cohort using scanned paper ECGs rather than digital signals; (ii) it incorporates variables not captured by CHA_2_DS_2_-VASc and demonstrates higher discrimination than clinical tabular features-only baselines in the same cohort; and (iii) it provides modality-level and feature-level interpretability, showing that scanned ECG paper images contribute additional signal beyond routine clinical data.

Prior analyses from the KERALA-AF registry focused on clinical predictors and had larger event counts, improving statistical power but they did not leverage image information.^12^, ^13^, ^14^^,^^32^ Taken together, our findings align with—and extend—the emerging evidence that multimodal deep learning can meaningfully enhance risk prediction over clinical data alone, particularly in resource-diverse settings where scanned paper ECGs are ubiquitous.

Several important limitations of this study must be emphasised. First, the sample size was relatively small, particularly for the positive (stroke) cases, which may limit model generalisability and increase the risk of overfitting. Although we restricted the analysis to 20 key variables to mitigate this issue, some potentially relevant predictors might have been omitted. Second, the precise timing of ECG acquisition relative to the exact phase of AF diagnosis or treatment status was not available in the dataset. This limits our ability to assess how specific clinical states at the moment of recording might influence the extracted ECG features. Third, due to the class imbalance, the discrimination performance of several machine-learning algorithms should be interpreted with caution, and external validation in larger and more diverse cohorts is warranted. Fourth, the multimodal deep learning framework adopted a simple late-fusion baseline that combines paper-based ECG features with clinical variables. The optimal model architecture, including fusion strategy, network depth, and parameter configuration, remains to be further explored. Furthermore, the analysis was restricted to a 1-year prediction window. As an ancillary analysis of the KERALA-AF registry, the study was bound by the parent protocol, which was specifically designed and funded to ascertain 1-year clinical outcomes as the primary endpoint. While this precludes the evaluation of long-term stroke risk, the primary objective here was to validate the feasibility of the multimodal deep learning architecture in a South Asian cohort. Finally, approximately 16% of the original cohort were lost to follow-up, which may introduce selection bias.

Integrating scanned paper ECGs with clinical data via deep learning methods significantly enhanced 1-year stroke risk prediction in South Asian AF patients. This study demonstrates the value of using multimodal AI with readily available, non-digital data in improving clinical risk stratification beyond current approaches based on clinical risk factors alone.

## Contributors

Qinkai Yu conceived and designed the study, developed the deep learning models, performed data analysis and statistical analysis, interpreted the results, and drafted the manuscript. Jinbert L Azariah contributed to data acquisition, data curation, and verification of the underlying clinical and ECG data. Z. Sajan Ahmad contributed to data collection and clinical data interpretation. Rajappan Anilkumar contributed to data collection and clinical input. Peter Calvert and Yang Chen contributed to data preprocessing, data analysis, and interpretation of the results. Yalin Zheng and Yanda Meng supervised the methodological development, provided critical input on study design and analysis, and revised the manuscript. Bahuleyan Charantharayil Gopalan and Gregory Y. H. Lip provided senior clinical oversight, contributed to study conception and interpretation of findings, and critically revised the manuscript. Qinkai Yu and Jinbert L Azariah had full access to and verified the underlying data. Qinkai Yu, Yanda Meng, Bahuleyan Charantharayil Gopalan, and Gregory Y. H. Lip were responsible for the decision to submit the manuscript. All authors reviewed and approved the final version of the manuscript.

## Data sharing statement

Data will be available on a reasonable request to the corresponding author.

## Declaration of interests

None of the authors declare competing interests.
